# Lipidomic-Based Advances in Diagnosis and Modulation of Immune Response to Cancer

**DOI:** 10.3390/metabo10080332

**Published:** 2020-08-14

**Authors:** Luis Gil-de-Gómez, David Balgoma, Olimpio Montero

**Affiliations:** 1 Center for Childhood Cancer Research, Children’s Hospital of Philadelphia, Colket Translational Research Center, 3501 Civic Center Blvd, PA 19104, USA; 2 Analytical Pharmaceutical Chemistry, Department of Medicinal Chemistry, Uppsala University, Husarg. 3, 75123 Uppsala, Sweden; david.balgoma@ilk.uu.se; 3 Spanish National Research Council (CSIC), Boecillo’s Technological Park Bureau, Av. Francisco Vallés 8, 47151 Boecillo, Spain; olimpio.montero@dicyl.csic.es

**Keywords:** immunotherapy, cancer, lipids, biomarkers, metabolism

## Abstract

While immunotherapies for diverse types of cancer are effective in many cases, relapse is still a lingering problem. Like tumor cells, activated immune cells have an anabolic metabolic profile, relying on glycolysis and the increased uptake and synthesis of fatty acids. In contrast, immature antigen-presenting cells, as well as anergic and exhausted T-cells have a catabolic metabolic profile that uses oxidative phosphorylation to provide energy for cellular processes. One goal for enhancing current immunotherapies is to identify metabolic pathways supporting the immune response to tumor antigens. A robust cell expansion and an active modulation via immune checkpoints and cytokine release are required for effective immunity. Lipids, as one of the main components of the cell membrane, are the key regulators of cell signaling and proliferation. Therefore, lipid metabolism reprogramming may impact proliferation and generate dysfunctional immune cells promoting tumor growth. Based on lipid-driven signatures, the discrimination between responsiveness and tolerance to tumor cells will support the development of accurate biomarkers and the identification of potential therapeutic targets. These findings may improve existing immunotherapies and ultimately prevent immune escape in patients for whom existing treatments have failed.

## 1. Introduction

Following the discovery of the structure of DNA in 1953 [1], increasingly efficient technologies for the study of the whole genome (genomics) have enabled assessments of genome-based pathologies in large population cohorts [2]. However, since a broad number of factors, including environment, diet or lifestyle, are important in the etiology of diverse diseases such as cancer, a high-dimensional biological approach appears to be required [3]. A multi-omics/systems-level approach, which encompasses the combined analysis of data from genomics, RNA transcription (transcriptomics), proteins/peptides (proteomics) and metabolites (metabolomics), enables one to overlay gene information onto a complementary understanding of accrued molecular mechanisms [4]. Lipidomics represents an emerging discipline from metabolomics that connects lipid biology, technology and medicine, and that strives to build an all-inclusive atlas of the cellular/tissue lipidome [5]. In this regard, the role played by lipids in the etiology and treatment of cancer has loomed large over the last decades.

**Figure 1:**
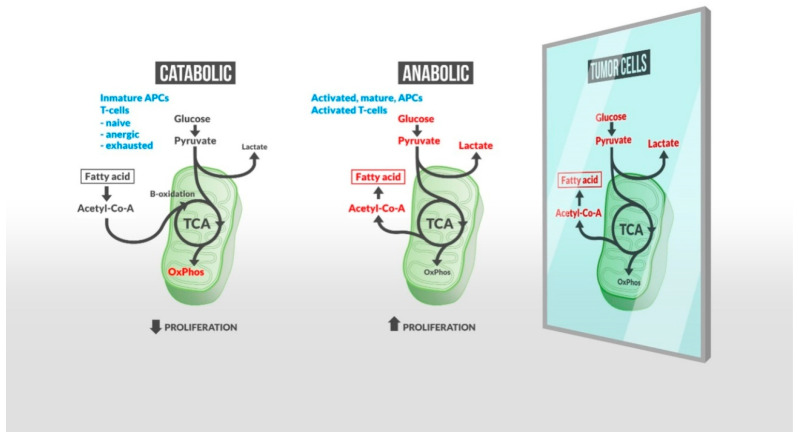
A metabolic shift is required by immune cells for them to respond actively to tumor cells. Inactive immune cells rely on oxidative phosphorylation (OxPhos) and fatty acid (FA) oxidation (left), while activated and responsive cells increase glucose uptake/glycolysis, resulting in an increased FA synthesis and lactate production (central panel). Lipogenesis, required for a robust cell proliferation, also characterizes tumor cell metabolism (right). Therefore, an untargeted lipid-based treatment to fuel effector immune cells may produce self-defeating effects inducing tumor cell growth. Many other lipid intermediates regulate inflammation, and exogenous lipids such as gut microbiota-derived short chain fatty acids (SCFAs) may impact the host immune response to tumor cells. Together, these findings indicate (1) the exhaustive regulation required to maintain immunity balance in the presence of tumor cells, and (2) the essential role of a large variety of lipids in this control. New precise lipidomic-based strategies may enhance therapeutic targeting and improve the capacity of existing immunotherapies to control tumor progression.

Early evidence that cancer cells undergo characteristic metabolic alterations was documented by Otto Warburg in the first half of the twentieth century. In a paradoxical process in terms of adenosine triphosphate (ATP) production, cancer cells increase the consumption of glucose to support aberrant cellular proliferation. Because proliferating tumor cells require cholesterol and other lipids, perturbations in the lipid metabolism are emerging as potential targets for therapeutic intervention in cancer [6,7]. Cancer immunotherapy has proven to have an unprecedented positive impact in clinical oncology. Increased evidence suggests that glycolytic metabolism not only rules cancer signaling but also the antitumor immune response where activated inflammatory immune cells display the same metabolic profile as tumor cells [8] (Figure 1). Multiple studies have separately reported the impact of lipids on immune cells and tumor progression. However, so far, little work has focused on reviewing how the lipid metabolism is associated with the immune response to tumors. Taking this shortfall into account, we aim to highlight the role of lipid mediators in the context of immune activation in order to explore potential biomarkers and therapeutic targets for cancer.

## 2. Lipid Metabolism Impacts Immune Activation against Tumor Progression

### 2.1. Lipid Interplay with Immune Regulation

Tumors impact immune cell function by supporting cancer stem cell survival, metastasis and immune evasion. The aggressiveness of tumor cells is linked to their capacity to store high levels of lipids and, in particular, cholesterol [6]. Metabolic challenges in the tumor microenvironment (TME), including hypoglycemia and hypoxia, induce changes in tumor cellular metabolism like aerobic glycolysis and fatty acid oxidation (FAO) [9]. In response, immune cells show the capacity to modulate lipid metabolism to better adapt to these special metabolic conditions.

The innate immune system is the first barrier against external stimuli, which are recognized via Toll-like receptors (TLR). TLR-dependent response, which regulates the activation of antigen-presenting cells (APC) (mainly macrophages or dendritic cells (DCs)), shifts the intracellular metabolism towards the glycolysis-fueled synthesis of fatty acid (FA) [10,11]. After the initial broad immune response, an adaptive immune response is initiated when APCs process and present antigens for recognition by certain lymphocytes such as T cells. Both phases of the immune response are characterized by a fragile equilibrium, whereas the heterogeneous groups of immune cells communicate and modulate each other via cytokine release. In this sense, cytokine production in activated DCs has been related to phospholipid remodeling to support FA demands [12]. Immune effector cells, such as T cells and macrophages, are induced by tumor-specific antigens and tumor-associated antigens. However, regulatory mechanisms of the immune system, such as immune checkpoints, make this cellular response incapable of preventing tumor progression. Immune check points are inhibitory regulators crucial for maintaining self-tolerance and controlling the duration of the immune response in order to prevent collateral tissue damage [13]. Since these key immune-regulatory molecules are used by tumor cells to promote evasion, immune checkpoint inhibitors have demonstrated their effectiveness as clinical targets for cancer immunotherapy [14]. This breakthrough is based on currently approved blocking monoclonal antibodies that inhibit cytotoxic T-lymphocyte-associated protein 4 (CTLA-4) and the programmed cell death protein PD-1/PD-L1 axis [15].

Endogenous lipid reserves provide energy to T cells but may also regulate T cell function by an immune checkpoint such as PD-1 [16]. PD-1 is a member of the cluster of differentiation 28 proteins (CD28) superfamily that delivers negative signals upon interaction with its two ligands, the PD-L1 and PD-L2 proteins. PD-1 activation impairs glucose and glutamine uptake whilst promoting FAO and catabolism of endogenous esterified fatty acids in both cytotoxic (CD8^+^) and helper (CD4^+^) T cells [16,17]. Another lipid pathway that targets PD-1 is regulated by the members of the peroxisome proliferator-activated receptors (PPAR) subfamily. This subfamily of nuclear receptors might be modulated by fatty acid signals derived from exogenous sources, including diet [18]. PPAR is crucial in supporting the accumulation and function of immunosuppressive regulatory T cells (Tregs) [19]. In concordance, it has been reported that PPAR-γ inhibition increases the efficiency of anti-PD-1 antibody immunotherapy, leading to the suppression of tumor progression in colon adenocarcinoma and melanoma models [20,21]; likewise, an agonist for another isomer, PPARα, is able to restore the anti-melanoma effects of tumor-infiltrating lymphocytes (TILs) by blocking the reprogramming to fatty acid catabolism in mice [22].

TILs, largely comprised of CD8^+^ and CD4^+^ T cells, as well as natural killer (NK) cells, are key players in tumor cell death. This particular function of both cell subtypes has been shown to be dependent on the profile of polyunsaturated fatty acids (PUFAs) in the cell membrane [23]. However, current work on how PUFA supplementation may affect TIL function in humans is often contradictory. Whereas it has been reported that the percentage of NK cells in mouse blood is reduced after dietary supplementation of docosahexaenoic acid (DHA, 22:6 n-3) and eicosapentaenoic acid (EPA, 20:5 n-3) [24], a similar previous study using EPA-rich oil in the diet did not find such differences [25]. The discovery of the G-protein-coupled receptors (GPCRs) suggests that many of the effects of dietary FAs may be receptor-mediated. This family of cell-surface free-fatty acid receptors includes the long-chain fatty acid receptors FFA1 and FFA4. Anti-inflammatory effects of omega-3 PUFAs, especially EPA and DHA, have been related directly to the expression of these FFA receptors. Hence, FFA4 knock-out mice have shown a higher proportion of pro-inflammatory macrophages than the wild type [26]. In addition, agonists of FFA receptors have been connected with the suppression of the proliferation and migration of a large variety of tumor cells [27,28].

The phenotype and maturation of T cells is also regulated by the fatty acid metabolism. Differentiation of T cells is dependent on de novo FA synthesis and uptake. In tumor tissue, the inhibition of de novo fatty acid synthase (FAS) by different targets, such as acetyl-CoA carboxylase 1, promotes Tregs but suppresses memory T cell lineage (Th17) differentiation [29]. The challenge of maintaining T cell function in a nutrient-depleted environment like the TME is resolved by other effector T cells. Unlike naïve and central memory T cells, effector memory T cells are less dependent on FA metabolism [30]. This feature plays an essential role in establishing immune equilibrium, since most effector T cells are removed after antigen elimination, whereas memory T cells remain for rapid response upon antigen re-exposure. The analysis of other molecules such as the mammalian target of rapamycin (mTOR) extends the list of lipid mediators that contribute to maintaining the immune balance. mTOR regulates Tregs differentiation, function and survival, ultimately defining the immunosuppressive profile of the TME [31]. Tregs are a dominant suppressive population that infiltrate the TME and dampen anti-tumor immune responses by inhibiting the effector T-cell function [32]. The singular metabolism of Tregs, including an increased FAO, provides them with critical advantages to survive and proliferate under hypoxia or low glucose conditions within the tumor [32,33].

The delivery and cellular distribution of PUFAs are indirectly regulated by desaturases, which perform the desaturation and elongation of essential fatty acids. However, phospholipases A_2_ (PLA_2_) are the main cellular regulators of PUFA release, maintaining the homeostatic levels of several free PUFAs, and in particular of those that are precursors of mediators with pro-inflammatory properties, such as arachidonic acid (AA, 20:4 n-6). In the inflammation process, AA is released by PLA_2_ activity, and prostaglandin E_2_ (PGE_2_) is subsequently generated from arachidonic acid by the enzyme cyclooxygenase-2 (COX-2) [34,35]. One of the mechanisms that Tregs uses to suppress T cell activity is PGE_2_ production, which can be reversed by COX-2 inhibitors [36]. PGE_2_ is essential in homeostasis, and while its pro-inflammatory role is crucial for host cell self-preservation, its immunosuppressive effects may support tumor progression [37]. Besides directly mediating inflammation, PGE_2_ might be used as an intermediate not only in the signaling between immune cells but also between immunity and tumors. Hence, PGE_2_ released from DCs affects the generation and proliferation of Tregs by immunosuppressive cytokines like IL-10, whereas PGE_2_ released from tumor cells is able to regulate DC maturation [37,38,39]. This COX2/PGE_2_ pathway is also involved in the regulation of the immune checkpoint enzyme expression, like PD-L1, in tumor-infiltrating macrophages and other myeloid cells [40]. Moreover, a recent study suggests that the combined blockade of PD-1 and PGE_2_ pathways is a promising therapeutic strategy for enhancing antitumor activity. This effect is due to an increased frequency of T cell-recognized tumor antigens, whose dysfunction is regulated by PD-1 [41].

Suppressing tumor immune surveillance may lead to the exhaustion or inactivation of pro-inflammatory immune cells and may, subsequently, promote tumor growth and metastasis. Myeloid-derived suppressor cells (MDSC) and immunosuppressive type II (M2) tumor-associated macrophages (TAMs) are fueled by the ß-oxidation of lipids, rather than glycolysis, within the TME [42]. Recent studies have shown that the phenotype of M2-like TAMs is controlled by intracellular long-chain fatty acid (LCFA) homeostasis, specifically unsaturated fatty acids like oleate [43]. Additionally, lipid metabolism provides a mechanistic explanation for TAM polarization and differentiation [44]. The upregulation of lipogenesis by sterol regulatory element-binding protein-1 (SREBP1) promotes the transcriptional response of macrophages to TLR signaling by driving the synthesis of anti-inflammatory fatty acids [45]. SREBP1 signaling also impacts tumor cells by sustaining the high energetic demands required for their growth and survival, and has been shown to be important in melanoma and prostate cancer progression [46,47,48,49]. One of the metabolic effects of SREBP1 is the regulation of the de novo lipogenesis by the upregulation of, among others, fatty acid synthase (FAS) and stearoyl-CoA desaturase-1 (SCD-1) [50,51]. Consequently, the upregulation of SREBP1 entails the upregulation of saturated and monounsaturated fatty acids, both free and in glycerolipids. Regarding macrophages, stimulation by lipopolysaccharide (LPS), a component of cell wall of gram negative bacteria, upregulates SREPB1 expression which is required for the inflammatory response [52,53]. In contrast, the activation of liver X receptors (LXRs), which also upregulate SREPB1, decreases the inflammation level in macrophages [54]. Because of this opposed effect, it is expected that the level of de novo lipogenesis in TAMs presents a complex relationship with their activation state. LXRs are regulated by oxysterols and SREPB1 by sterols in the cell environment. Consequently, not only diet but also the tumor lipid microenvironment can regulate the metabolic/pro-inflammatory status of TAMs. In addition, external palmitic acid reprograms the microglia metabolism in a way that mimics LPS treatment [55], whereas oleic acid reduces the pro-inflammatory response [56]. Furthermore, sexual hormones in the TME also play a key role in the lipid metabolism and the inflammatory state of TAMs. The androgen receptor decreases the LXR and SREBP1 activity, which decreases the de novo lipogenesis and remodels the lipid metabolism [57,58]. Interestingly, the interaction between prostate cancer cells and macrophages regulates the resistance to hormonal therapy [59]. This fact suggests an interplay in tumor growth among: (1) the activation of the androgen receptor, (2) the tumor microenvironment and (3) the LXR-mediated lipogenesis in both the tumor and TAMs. Altogether, these studies suggest that both the lipidic and hormonal microenvironment interact to reprogram the metabolic and inflammatory state of TAMs. This reprogramming is associated with therapy resistance and patient prognosis.

LXRs are major regulators of FA and cholesterol homeostasis. Cholesterol, a nonpolar lipid transported in plasma by low-density lipoproteins (LDL) and high-density lipoproteins (HDL), has been linked to the effect of IL-10 in immune regulation. The inhibition of cholesterol biosynthesis with atorvastatin or 25-hydroxycholesterol regulates IL-10 production by inducing human CD4^+^ T cells to switch from an effector to an anti-inflammatory profile [60]. Furthermore, given the role of lipoproteins as cholesterol carriers, while they promote tumor growth by regulating T cell activation and functionality [61], recent studies have used them as anti-tumor drug delivery vehicles [62].

The impact of lipids on the immune response to cancer includes post-translational modifications. Palmitoylation has been found to be important in the context of cancer immunotherapy. This post-translational process involves the binding of palmitate (C16:0) to amino acid residues. Yao and colleagues identified palmitoyl transferase ZDHHC3, which contains a conserved Asp-His-His-Cys (DHHC) signature motif, as the main acyltransferase required for PD-L1 palmitoylation. This lipid modification stabilizes PD-L1 by blocking ubiquitination, which ultimately prevents lysosomal-driven degradation. Thus, DHHC3 targeting enhances T cell cytotoxicity against cancer cells in vitro, as well as the in vivo antitumor effect in a colon carcinoma model [63]. Other studies have related the ablation of ZDHHC3 in human mammary tumor cell xenografts to a reduced primary and lung metastasis infiltration. This effect correlates with an enhanced recruitment of macrophages and NK cells to the tumor, and its subsequent clearance [64].

### 2.2. Short-Chain Fatty Acids from Gut Microbiota as Effectors of the Immune System

FAs with chain lengths ranging from one to six carbon atoms are produced by trillions of harmless microorganisms that inhabit the human gastrointestinal tract. These short chain fatty acids (SCFAs) are the major end product derived from gut microbiota; very high concentrations are found in the colon [65]. The presence of SCFAs (propionic, butyric, acetic and valeric acids) regulates the intestinal microenvironment by reducing pH and impacting the microbial function and composition [66]. Besides various gut disorders, gut microbiota also play an important role in central nervous system disorders, the immune system and cancer malignancies [67]. Although the role of butyrate in fueling tumor cells proliferation has been described [68], SCFAs have been generally perceived as tumor suppressors because they induce cancer cell differentiation and apoptosis [69]. The ability of SCFAs to regulate effector immune cells is considered one of the essential mechanisms accounting for their anti-tumor properties [70]. SCFAs engage GPCRs such as FFA2 and FFA3, and act as histone deacetylases (HDACs) to regulate the activity of innate immune cells such as neutrophils, macrophages and DCs, and they also modulate antigen-specific adaptive immunity mediated by T cells and B cells [71,72].

SCFAs, particularly butyrate, directly impact the immune response to cancer through the reprogramming of the cellular metabolism. In activated CD8^+^ T cells, butyrate increases glycolytic activity, mitochondrial mass and membrane signaling. Butyrate-stimulated CD8^+^ T cells also show functional uncoupling of the TCA cycle from glycolysis, promoting additional sources of carbon such as glutamine and FAs [73]. An increased FA intake in butyrate-treated CD8^+^ T cells serves to charge the TCA cycle, but triacylglycerides and phospholipids are other candidates that serve as suppliers [74,75]. The anti-inflammatory properties of SCFAs are also related to the ability of butyrate and propionate to abrogate IL-12 release from APCs, a cytokine with a primary role in effector T cell stimulation [76,77]. In contrast, both SCFAs are also associated with resistance to immune checkpoint CTLA-4 blockade and a higher proportion of Treg cells. These effects limit the clinical outcome of cancer patients treated with anti-CTLA-4 blocking monoclonal antibodies [78].

The capacity of butyrate to regulate T cell polarization and immune checkpoint blockade correlates with the diversity of commensal microbiota. In human bacterial communities, most butyrate-producing colon bacteria belong to the *Firmicutes* phylum. The equilibrium between species defines the therapeutic outcome, and a low Bacteroidetes/Firmicutes ratio has been used to identify lung cancer patients [79]. Moreover, the relative abundance of other specific bacteria, such as Bifidobacterium, increases anti-PD-L1 efficacy, promoting anti-tumor immunity [80]. Taken together, these findings point toward an alternative therapeutic strategy by targeting immune cells on a metabolic level. Augmenting the efficacy of the immune system by targeting the lipid metabolism could be useful for improving the antitumor immune response. However, as Chalmin et al. postulate, targeting the lipid metabolism may affect multiple immune populations and could have unpredictable outcomes [81]. Thus, since fatty acid oxidase is required not only for effector T cell development but also for Treg differentiation [82], its blockade limits Treg-dependent immunosuppression. Despite these drawbacks, data suggest that the capacity to define specific lipid reprogramming that correlates with disease stages will help to design new cancer treatments. The balance between immune activation and suppression is a critical feature of immunity, and lipids are able to alter this equilibrium. Therefore, targeting the lipid metabolism may be used to induce immune stimulation, which will ultimately determine the clinical success of cancer immunotherapy.

## 3. Lipids as Biomarkers of Immune Response to Cancer

Accurate and predictive biomarkers to diagnose early stages of disease are a critical objective of clinical and biomedical research. Lipids, among several other metabolites such as amino acids or sugars, have been described as potential predictors of systemic alterations that discriminate between healthy controls and patients [83]. Clinical success often hinges on an early diagnosis, especially in long and age-related malignances like Alzheimer’s disease or cancer [84]. New technologies for the qualitative and quantitative analyses of metabolites can provide essential information on pathological conditions that can result in profound alterations in the architecture of the immune system. Identifying the metabolic profile associated with the immune response to tumor cells has emerged, parallel to immunotherapy, as a tool for obtaining an early and accurate diagnosis and for designing personalized treatments, both being essential for better clinical outcomes in cancer patients.

An increased de novo synthesis of fatty acids is required for membrane synthesis and, therefore, for the growth and proliferation of both immune and tumor cells. This makes fatty acids robust biomarker candidates. Recent studies have shown that genetic alterations observed in acute myeloid leukemia (AML) patients control lipid dynamics and metabolism [39,85]. Interestingly, patients with AML can be identified by specific lipid signatures in plasma [86] and bone marrow [87]. Whereas lipid biomarkers have been used to identify tumor progression, the relationship between a characteristic lipid profile and the immune response to cancer is still poorly understood. The major clinical advantages of immune checkpoint inhibitors have generated considerable interest in discovering biomarkers that predict the response to treatment [88]. Recent studies propose serum concentrations of very long chain fatty acids (VLCFA) as a way to identify the response to immune checkpoint inhibitors in urological cancer [42]. The rationale for this biomarker is motivated by the finding that lower serum VLCFA levels are associated with highly immunosuppressive TME with a high-VLCFA consumption rate.

As discussed previously, de novo lipogenesis is also associated in a complex manner with the metabolic/inflammatory state of TAMs. Consequently, the lipids associated with the de novo lipogenesis act as biomarkers of tumor growth and the activation of TAMs. The LXRs/SREBP1 pathway is the key player in the regulation of the de novo lipogenesis, and it is involved in tumor growth and in the inflammatory response [89]. LXRs/SREBP1 upregulation in tumor or inflammatory cells leads to an increase of saturated and monounsaturated fatty acids via the activation of FAS and SCD-1, which are incorporated into glycerolipids by acyltransferases. Consequently, the upregulation of glycerolipids with saturated and monounsaturated fatty acids acts as a biomarker for the tumor synthesis of membranes and the activation of macrophages [90,91]. In addition, LXRs/SREBP1 upregulate glycerol-3-phosphate acyltransferase 1 (GPAT-1), which has a strong preference for transferring palmitic acid to the *sn*-1 position of glycerol-3-phosphate. This leads to an enrichment of glycerolipids with palmitic acid in the *sn*-1 position of the glycerol backbone. Consequently, the triacylglycerides with palmitic acid in the external position of the glycerol act as a biomarker of LXRs/SREBP1 activation and the de novo lipogenesis [92,93]. In conclusion, these triacylglycerides have the potential to be used as biomarkers for (1) monitoring the metabolic reprogramming of TAMs in the TME, and (2) the effect or resistance to immunotherapy by evaluating the up- or downregulation of lipogenesis in the tumor.

Because SCFAs from gut microbiota have a wide-ranging impact on the host physiology, these metabolites are also increasingly studied as predictive biomarkers. SCFAs and microbiota composition have been used to determine the risk of cancer, and reduced levels of butyric acid in patients with colon cancer have been reported [94,95]. The levels of butyrate are also correlated with the responsiveness to melanoma in mice treated with antibiotics [96]. Recent results have reported a correlation between the relative abundance of certain SCFA-producing microbiota and the outcome of PD-1-based immunotherapy in melanoma patients [97]. These data correlate with those from a recent study that makes the case for the reduced serum content of SCFAs being a biomarker of refractory non-small cell lung cancer (NSCLC) [67]. According to Boticcelli et al., lower levels of SCFA are found in the fecal samples of patients with a poor prognosis treated with Nivolumab, a human PD-1-blocking antibody. Together, these results show that gut microbiota-induced immune effects are dependent on the specific cancer therapy and that certain blood lipid biomarkers are able to predict this relationship.

When cancer care is delayed, patient treatment is associated with greater clinical complications and a lower survival rate. In order to have the best chance for a successful treatment and prognosis, an early and precise diagnosis of cancer progression before and during the treatment is critical. Thus, identifying biomarkers that can monitor the tumor response in every stage of treatment has huge clinical implications. Further studies will be needed to correlate the lipid profile with the immune cell phenotype and immune checkpoint expression within the tumor. These data will help to discriminate between pro-inflammatory and immunosuppressive TME populations, resulting in more accurate biomarkers of cancer progression.

## 4. Active Modulation of Lipid Metabolism to Improve CAR T Cell Therapy

Chimeric antigen receptor—engineered T cell (CAR T) therapy has demonstrated its long-term clinical benefit for patients with advanced cancers [98]. CART therapy involves genetically modified patient T cells with chimeric antigen receptors that recognize specific antigens on the tumor cell surface. The antitumor efficacy of immunotherapy against hematologic cancers has been extended to other tumors [99,100,101]. Among diverse potential targets, such as CD19 for B-cell malignancies, GD2, a disialoganglioside glycolipid, was identified as a tumor antigen more than 30 years ago [102]. GD2 is normally present in developing brains and can be overexpressed in some tumors, with a greater recurrence in childhood cancer neuroblastoma, melanoma and diverse pediatric sarcomas [103]. However, while GD2-specific antibody therapies used in the treatment of neuroblastoma have been shown to be successful, the fatal neurotoxicity of GD2-specific CAR T cell therapy that has been observed in some studies suggests that GD2 may be a difficult target antigen for CAR T cell therapy [104].

Several studies and clinical trials reveal that CAR T cell therapy for leukemia achieved high rates of complete remission, but therapy-relapsed leukemia remains a significant source of mortality [105]. Because T cell exhaustion elevates the risk of relapse [106], additional research on how to avoid this detrimental effect is urgently needed. Differentiated effector T cells use glycolysis for proliferation, and after activation they ultimately succumb [21]. Only a small proportion of long-surviving memory T cells with OXPHOS-mediated ATP production contributes to a favorable and durable antitumor response in the TME [107]. Since Notch signaling, a conserved cellular interaction mechanism, promotes mitochondrial biogenesis and FA synthesis, recent studies have evaluated the impact of the manipulation of this metabolic pathway on the success of CAR T cells. According to Kondo et al. [108], the overexpression of Notch and its downstream gene Forkhead box M1 (FOXM1) results in enhanced anti-tumor effects as compared with conventional CART cells, suggesting a novel strategy to improve CART-based therapy [108].

The success of CAR T cell therapy in treating hematological malignancies is limited in solid tumors, where finding, entering and surviving in the tumor are extra challenges [109]. Other restrictions are driven by constraints from the on-target off-tumor toxicity of CAR T cells, where the lack of tumor specificity increases the potential risk for normal tissues to be attacked by CAR T cells [109,110]. In order to avoid these limitations, new strategies have focused on providing an anti-tumor effect with an absence of side-effects. Besides the enormous ability of PUFAs, such as AA, EPA and DHA, to regulate the immune responses, as presented above, gamma-linolenic acid (GLA, 18:3 n-6) has shown a selective effect against tumor cells [111]. According to an open-label clinical study that included 21 patients with stage IV glioma, the intra-tumor injection of GLA enhanced the sensitivity of tumor cells to chemotherapeutic drugs and radiation, producing tumor regression without harming normal cells [112]. Additionally, together with AA, EPA and DHA, GLA has been reported to regulate the antioxidant properties of glutathione peroxidase 4 (GPX4), as well as the levels of cytokines such as IL-1, IL-6 and tumor necrosis factor alpha (TNF-α) that play essential roles in inflammation [113,114]. These data suggest that the combination of PUFAs as an adjuvant may help immunotherapy block tumor progression.

Lipid metabolism has a dual impact on CAR T cell therapy. Lipids can systematically fuel tumor cells and immune cells. However, enhancing the immune response via CAR T cells presents evident advantages beyond the described obstacles. T cells can be successfully designed and prepared for the restricted metabolic conditions within the TME [115]. Identifying and reprogramming the mechanisms involved in the dysfunction of CAR T cells may help support more proliferative and ultimately successful CART cell-based therapies [109]. Therefore, metabolic targets that include the lipid metabolism may generate improved CAR T cells, so as to avoid cancer relapses related to T cell disability.

## 5. Conclusions

The complexity and variability of tumors still constitute a challenge for physicians and researchers. Although immunotherapy has attained ambitious milestones and improved prognoses for cancer patients, the systemic character and self-regulation capacity of immunity should be considered in order to obtain improvements. A multi-focal anti-tumor strategy, in combination with other treatments, appears to be required in order to avoid relapses; moreover, it should draw from diverse perspectives: First, a global intervention, by for instance modulating the gut microbiota, which could have positive effects on the immune cell activity; second, an early and precise diagnosis so as to achieve better clinical outcomes; and third, targeted treatments, where genetically engineered patient CAR T cells have already shown clinical benefits.

Current treatment limitations are related to an immunosuppressive TME, which modifies the T cell function in terms of differentiation and exhaustion. Combining CAR T cells with checkpoint inhibitors and the depletion of suppressive factors in the microenvironment via lipid targets may mitigate this phenomenon. Although new studies will be necessary to characterize specific metabolic pathways implicated in the immune response to tumor cells, data suggest that lipid reprogramming will be key to generating a favorable metabolic environment to avoid tumor evasion.

In conclusion, the modulation of the immune system has been extensively demonstrated to be an effective cancer treatment. However, further investigations should focus on reducing treatment limitations that ultimately lead to tumor relapse. Several studies are currently focusing on therapy improvements by facilitating energy influx to T cells, where lipids play an essential role. Targeting lipid reprogramming in the immunity setting may generate new tools to create lasting, robust and personalized therapies against cancer.
